# Community of Practice of *Promotoras de Salud* to address health inequities during and beyond the COVID-19 pandemic

**DOI:** 10.3389/fpubh.2023.1260369

**Published:** 2023-11-13

**Authors:** Patricia Rodriguez Espinosa, Yessica Martinez Mulet, Wei-ting Chen, Cary Kirk, Cindy Tran, Mike Gonzalez, Lisa G. Rosas, Promotoras Con Stanford En Acción

**Affiliations:** ^1^Department of Epidemiology and Population Health, Stanford University School of Medicine, Palo Alto, CA, United States; ^2^Office of Community Engagement, Stanford University School of Medicine, Palo Alto, CA, United States; ^3^Office of Patient Experience, Stanford Health Care, Palo Alto, CA, United States; ^4^Santa Clara Family Health Plan, San José, CA, United States; ^5^Community Coalition of Promotoras, San José, CA, United States

**Keywords:** COVID-19, health equity, Latinx health, community health workers, health promotion, capacity building

## Abstract

Using principles of Community-Based Participatory Research, we describe a community of practice for community health workers and promotoras (CHW/Ps) to address COVID-19 inequities in the Latinx community. We offer a concrete example of how programs can engage CHW/Ps as full partners in the research process, and how programs can support CHW/Ps’ capacity and workforce development during implementation. We conducted four focus groups with CHW/Ps (*n* = 31) to understand needs and invited 15 participants to the community of practice to work on issues identified by the group. We examined impact according to number of community members reached, types of outreach activities, surveys, and online views of educational materials. Process evaluation involved two focus groups with seven organizations and a Ripple Effects Mapping session with the CHW/Ps. Our community of practice has built CHW/Ps’ capacity via 31 workshop and co-created culturally and linguistically relevant COVID-19 materials that have reached over 40,000 community members and over 3 million people online. The community of practice proved effective in supporting CHW/Ps to address COVID-19 inequities in the Latinx community. Our evaluations demonstrated benefits for community-academic partnerships, for CHW/Ps, and for the community. This model represents an innovative workforce training model to address health inequities and can be applied to other health topics.

## 1. Introduction

The COVID-19 pandemic brought to the forefront severe long standing and systemically rooted inequities in burden of disease and mortality and in lack of access to medical and other resources among racial/ethnic minorities and other minoritized populations (1–3). Moreover, for many populations, especially Latinx and non-English speaking communities, the intersectionality of these inequities with lack of access to technology, language barriers, overrepresentation in high-risk occupations and other factors further complicated the response to this public health crisis (4, 5). Partnerships with trusted messengers, such as community health workers and promotoras (CHW/Ps), became a critical strategy for overcoming these systemic and nuanced challenges.

CHW/Ps, also known as *promotoras de salud*, health navigators, and community leaders are a crucial workforce well positioned to address inequities in medically underserved and marginalized communities. Programs that involve CHW/Ps have ranged in disease focus (e.g., chronic disease prevention, cancer care, mental health), community of focus (e.g., rural, urban, racial/ethnic minorities), and program goals and activities including outreach, capacity building, community education, informal counseling, social support, health care navigation, and advocacy (6–8).

While studies have shown that CHW/Ps are effective in serving underserved populations, including racial/ethnic minorities and low-income communities, and in improving a variety of health outcomes (9), most studies utilize CHW/Ps primarily in the delivery of interventions (9, 10), to connect participants with services, or as case managers (e.g., to promote treatment adherence) (11). Less is known about studies or programs that engage CHW/Ps as full partners in the research process, how these studies or programs support CHW/Ps’ capacity and workforce development, and the types of support that are provided to CHW/Ps during program implementation.

The goal of this paper is to describe our Community-Based Participatory Research (CBPR) approach (12, 13) in the development and implementation of a CHW/P community of practice to provide resources, support, and capacity building. We describe activities and lessons learned, evaluation efforts, and scaling plans.

## 2. Context

During the COVID-19 pandemic, there was a large influx of resources for CHW/Ps, such as funds allocated from Public Health Departments, and the National Institutes of Health Community Engagement Alliance sites (14, 15), to local community-based organizations (CBOs) with CHW/P networks (16, 17). This included CBOs whose CHW/Ps pivoted from a focus on diverse topics such as domestic violence, cancer, and nutrition, to a focus on COVID-19. While CHW/Ps were being relied on as trusted messengers to disseminate COVID-19 information and resources in their local communities, they did not have the necessary training or ongoing support to address the constantly changing community needs resulting from the pandemic. For example, CHW/Ps did not have the training to discuss intricacies of vaccine development, pandemic-related trauma, or evidence-based tools to address vaccine hesitancy with community members. This presented a partnership opportunity to enhance CHW/Ps’ capacity while supporting community outreach and education to reduce health inequities.

### 2.1. Population and recruitment

CHW/Ps from local CBOs were recruited for focus groups (*n* = 4) between December 2020 and January 2021 to assess their needs for best serving their communities during the COVID-19 pandemic. Local CBOs who employed CHW/Ps (e.g., faith-based, civic engagement, food or housing justice, health organizations), were sent an invitational flyer to share with their CHW/Ps. Focus group were conducted via Zoom and audio recorded for data analysis purposes. Consent was obtained via phone by a research coordinator before the focus groups, followed by email with the consent for signature. Verbal consent was obtained again at the start of the focus groups. All focus group participants were compensated with a gift card. Focus groups (*n* = 31 CHW/Ps) revealed the following overarching themes: (1) need for outreach in the Latinx community with culturally and linguistically appropriate materials, and (2) a need for capacity development and ongoing support for CHW/Ps engaged in COVID-19 outreach. CHW/Ps described the need for factual and evidence-supported educational materials that were developed for the Latinx community (e.g., utilizing colloquial sayings, emphasizing cultural values, and that considered literacy and language barriers). They also described the need for updating materials as the pandemic evolved and requested dissemination support (e.g., printing, support for their time, identifying key neighborhoods for outreach). Capacity development needs also emerged in all focus groups. CHW/Ps described their organizations being overwhelmed by direct provision of services and attending to needs exacerbated by the COVID-19 pandemic (e.g., housing and food insecurity), leaving them with little time or resources to devote to developing expertise in a new area beyond their direct organizational mission or services. Capacity development needs were identified around several areas, including the science of vaccine development, clinical trial basics, motivational interviewing for addressing vaccine hesitancy, mental health basics to address increasing community concerns around trauma, isolation, and stress related to COVID-19.

CHW/Ps (*n* = 15, 93% female, 100% Latinx individuals, primarily Spanish speaking) were then recruited from the focus groups according to interest in joining a community of practice to work on the issues discussed in the focus groups, and who represented community-based organizations with different missions (e.g., faith-based, civic engagement, health).

### 2.2. Setting

Santa Clara County, with a population of over 1.8 million (25% Latinx, 40% foreign born, 54% speak a language other than English at home) was the primary setting for recruitment and community outreach (18). Although Latinx individuals only represented 25% of the population, in 2021 they accounted for over 50% of new cases. In 2023, they accounted for over 31% of COVID-19 cases (only overrepresented racial/ethnic group) and over 30% of COVID-19 related deaths (19).

### 2.3. Evaluation metrics

We collected impact metrics for each of our main activities including: (1) co-development of culturally relevant materials (# of assets developed and their dissemination, such as # of individuals reached, # of website views); (2) capacity development via post-workshop surveys assessing relevance, uniqueness, culturally and linguistically appropriateness, and usefulness of the content; and (3) direct outreach (e.g., # of individuals reached, zip codes, types of events, which were entered by the CHW/Ps using an online form). Moreover, we collected data on process, including two focus groups with 23 CHW/Ps, representing 7 different organizations, along with five CBO leaders (*n* = 28 total attendees) assessing the usefulness of co-developed materials and any modifications needed. Recruitment centered on existing CBO partners utilizing the materials and new partners including the Mexican consulate who disseminated outreach materials to the community. Consent, compensation, and facilitation mirrored the initial focus groups described previously. To assess the community of practice process more generally, we employed Ripple Effects Mapping (REM) (20), a participatory evaluation method that assesses multi-level intended and unexpected impacts (e.g., for the CHW/Ps themselves, their organizations, the community) of a program –in this case capacity building and participation in the local CEAL project-, challenges and participant-identified solutions to those challenges. Qualitative data (from focus groups and REM sessions) were analyzed using thematic analysis (21) and lightening report methods for rapid qualitative synthesis involving identifying positive elements, suggestions for improvement, and future directions (22). NVivo software and Xmind software (23) were used for analysis.

## 3. Key programmatic elements

### 3.1. Developing the community of practice

Throughout the project, we adhered to best practices of CBPR including decision-making processes that ensured all partner’s voices are heard, budget and resource sharing, and centered on developing activities that directly addressed needs identified by the CHW/Ps. Building from existing community engagement efforts, the 15 Spanish speaking CHW/Ps recruited to join the community of practice started meeting bi-weekly to work on issues identified in the focus groups. Initial meetings were used to further develop objectives, understand each organizations’ strengths and resources, and develop processes for the community of practice. As the project progressed, meetings were also used to: (1) co-develop, and discuss feedback on materials developed for community outreach and education around COVID-19 (e.g., bilingual educational materials for the community); (2) to continue hearing community needs reported to the CHW/Ps and use the information to revise goals and activities; and (3) to engage in trainings and capacity building, described in further detail below.

**Figure 1:**
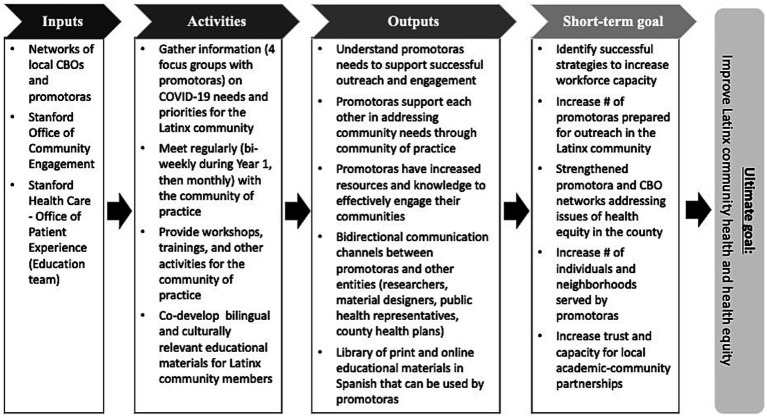
Logic model. CBO = community-based organizations.

The CHW/Ps community of practice decided to name themselves Promotoras con Stanford en Acción, and grew to be widely recognized in the community and local county for their efforts to address CHW/Ps’ workforce needs and for disseminating resources and information. Additional CBOs, county public health leaders, and leadership from the Santa Clara Family Health Plan, a local health payor, partnered with Promotoras con Stanford en Acción and provided both input, resources (e.g., staff time, physical meeting space, in-kind resources), and have joined in outreach and training efforts. The community of practice also served as a regular place to share resources and information, and to develop strong networks among the CHW/Ps and their respective organizations. See Figure 1 for a Logic Model.

### 3.2. Main activities

#### 3.2.1. Co-development of culturally and linguistically relevant educational materials

**Figure 2:**
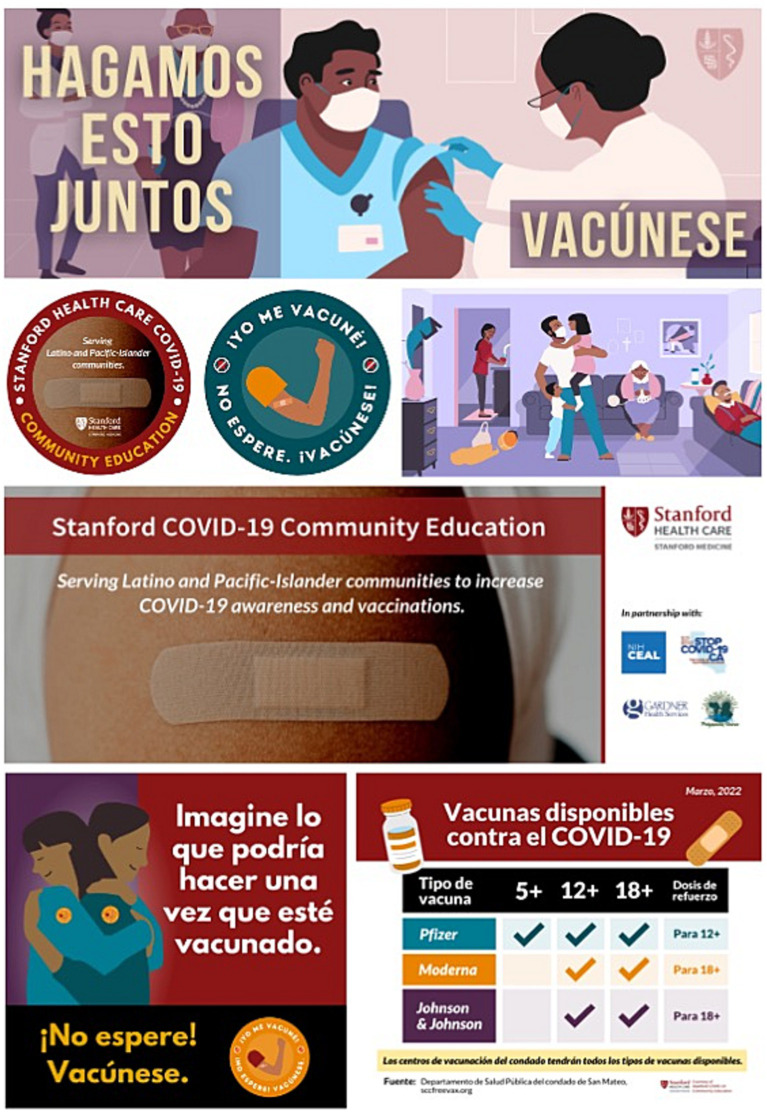
Sample of culturally and linguistically relevant educational materials.

In partnership with a patient education and web developer team from Stanford Health Care, we co-developed health education materials that included compassionate, engaging, culturally and linguistically relevant content. Through a five-step process including research, planning, development, dissemination, and evaluation, we co-created and distributed two websites in Spanish with content focused on the COVID-19 vaccines and Staying Safe, which centered on public health guidelines such as masking, quarantine, testing, and others. We also hosted 4 Spanish social media channels (Facebook, Instagram, Twitter, and YouTube) to store and share 47 social media assets (e.g., gifs, banners, thumbnails), 26 flyers, 2 posters, and 8 Public Service Announcement videos (see Figure 2). CHW/Ps provided vital insight into the needs, questions, and myths surrounding COVID-19 from the community, which were used in the development of the materials. The input from CHW/Ps and their respective organizations also served to inform formatting and dissemination strategies and ensured that the materials reflected the community needs. The final products were a result of an ever-evolving process that involved input from our CBOs, CHW/Ps, as well as being influenced by the changing COVID-19 landscape and our community’s needs (e.g., testing, vaccines, Long-COVID, mental health impacts).

Materials were disseminated online (via partner CBOs pages, our own pages, and via paid Facebook campaigns), at in-person events (e.g., health fairs, schools, markets), and during door-to-door and other outreach conducted by the CHW/Ps. For this effort we leveraged additional partnerships with new and existing CBOs, including non-profit agencies, community resource centers, local safety net clinics, and the Mexican Consulate Ventanillas de Salud program. Dissemination was conducted in areas with higher percentage of Latinx and other racial/ethnic minority groups (determined using Census tract information and local Public Health Department data), as well as high proportions of COVID-19 cases. Our CBO partners were predominantly agencies working with medically underserved and other marginalized communities.

#### 3.2.2. Capacity building

Emphasizing CBPR principles around capacity development (24) to enhance infrastructure, and contribute to long-term maintenance and sustainability, we developed a series of training sessions in a variety of topics (see Table 1) including COVID-19 science and latest developments, motivational interviewing for addressing vaccine hesitancy, and mental health basics (e.g., identifying symptoms, referring, stress and coping strategies, and addressing burnout among the CHW/Ps). These capacity building sessions often involved outside speakers including Stanford faculty and staff, as well as community representatives in a particular area of interest (e.g., mental health). We extended these trainings to other partners as well. For example, we conducted the motivational interviewing training, adapted for vaccine hesitancy concerns, for CHW/Ps and field workers working with the local Public Health Department. Since, we have extended trainings to CHW/Ps in our three local counties via ‘live’ online workshops.

**Table 1:** Evaluation metrics across activities.

Activity	Outputs	Process evaluation	Impact evaluation
Co-development of bilingual and culturally relevant educational materials	8 short PSAs focused on vaccine hesitancy, availability, and motivating community members to be vaccinated	Two focus groups (*n* = 28, 23 community health workers representing 7 different organizations, and 5 staff members) Lightening report with findings disseminated with partners and design team. Changes made to materials based on feedback: Increased emphasis on infographics and flyers. Social media toolkit developed for partner use in their organizations’ pages.	Paid Facebook campaigns on Spanish short PSAs (n = 3): 3.3 million people reached Over 11 million views and impressions 1764 views across websites and social media platforms (i.e., Instagram, Facebook, Twitter, and YouTube)	28 one-pagers or flyers with general vaccine information (eligibility, availability, side effects, etc.) and debunking common myths 47 social media assets including GIFs and images (e.g., social media thumbnails and banners)	32,000 views across communication and social media channels (e.g., Instagram, Twitter, Website, Text Messaging, etc.) Over 1700 website visits Disseminated to 3,500 community members via additional partners at in-person events
Workshops and capacity development trainings	Series of motivational interviewing workshops (*n* = 5) 3 within the community of practice 2 conducted for the Santa Clara County Public Health Department promotoras and field workers Mental health workshops related to basic emergency mental health response, coping strategies (e.g., self-care, mindfulness practice), stress, and burnout (*n* = 3) COVID-19 trainings (*n* = 23) including vaccine approval process, clinical trial basics, science of COVID-19 and vaccines, overview of vaccine eligibility and guidelines, etc.		Ripple Effects Mapping session (*n* = 15 promotoras) revealed usefulness of the trainings “learned how to give the information in a concrete way and that they feel that they can relate to me, like we learned here” “I wished I had this information before” “I feel ready to give the information to my community”	Motivational interviewing workshops specific sample items: 94% agree empathy and empowerment are key components of motivational interviewing 94% agree open-ended questions and reflection are key skills of motivational interviewing All workshops*: 96% agree the workshop content will improve their ability to work with the community 77% endorsed the content was new 96% agree the content was linguistically and culturally appropriate 92% agree they feel confident using and applying skills to daily work
Outreach activities	Door to door canvasing in hard-hit zip codes provided education, testing, and vaccination resources In zip codes most impacted by COVID-19 (e.g., 95116, 95127, 95122) Outreach in community settings: E.g., schools, community centers, parks, and food banks.	Ripple Effects Mapping session Challenges: safety, weather, myths and disinformation Solutions: peer-support, use of open-ended questions, sharing personal experiences to increase rapport, connecting families with basic needs to increase trust	40,634 community members reached between July 2021 and June 2023 In over 50% of reporting periods promotoras highlighted speaking with Latinx parents Within the Latinx community, promotoras reported interacting with essential workers, seniors, and with adolescents and children

*Calculated by averaging across all workshops and respondents.

#### 3.2.3. Community outreach and engagement

Utilizing data from the Public Health Department dashboard, which highlighted census tracts with high concentration of cases, mortality, and low vaccination rates (when vaccines became available), as well as high concentration of poverty and other structural factors, CHW/Ps conducted outreach in communities, particularly Latinx communities, overburdened by the pandemic. Using our co-developed materials, outreach activities aimed to decrease disparities by connecting community members with COVID-19 resources (e.g., testing, vaccination, education around worker rights pertaining to quarantine and time off due to COVID-19, helping comply with public health guidelines) and with basic needs resources (primarily housing and food) to address social determinants of health issues exacerbated by the pandemic.

The CHW/Ps conducted outreach using a multi-method strategy including door-to-door canvasing, when in-person interactions were possible, outreach at community events, health fairs, tabling at supermarkets, schools, community centers, and other locations. During outreach activities, CHW/Ps also kept track of key questions and myths brought forward by community members. These informed our development and revisions of educational materials throughout the project.

### 3.3. Evaluation metrics

Evaluation metrics included assessment of the main activities: (1) co-development of linguistically and culturally relevant COVID-19 education and outreach materials and their dissemination, (2) capacity development for the CHW/Ps, and (3) outreach activities in the community (Table 1).

#### 3.3.1. Process evaluation

Focus groups evaluating educational materials revealed overall cultural and linguistic acceptability of materials. Participants also shared that university branding enhanced credibility and trust during their outreach. Feedback informed further enhancements and updates, for example, stronger emphasis in developing single page infographics and flyers that could be more easily updated with ongoing feedback from the CHW/Ps (e.g., with new myths or changing guidelines). Moreover, partners requested social media posts and GIFs to be used by their organizations’ pages to address COVID-19 questions and myths from our local community members.

Findings from the Ripple Effects Mapping session (*n* = 15 CHW/Ps), facilitated by an outside researcher to the community of practice (LGR), revealed outcomes in several key areas as a direct result of participation in the community of practice: (1) new and strengthened social connections among the CHW/Ps themselves and their respective organizations, with academic researchers, and with communities; (2) personal benefits to the CHW/Ps such as increased skills, job satisfaction, and access to resources; and (3) perceived positive impact on the community (e.g., increased trust and connection, information and resources distributed, stories of positive impacts shared by community members). CHW/Ps also shared challenges including widespread myths and disinformation around COVID-19, refusals from community members in terms of testing or vaccination, and challenges in conducting door-to-door outreach (e.g., weather, safety).

#### 3.3.2. Impact metrics

Education materials had over 32,000 online views through the various channels (e.g., YouTube, Instagram, website). These materials were also disseminated in person by partner organizations to 3,500 individuals. In addition, a paid Facebook campaign (ran for 1 month in May 2021) results in 3.3 million individual views of the PSA videos. On average, the cost was $8.6 per 1,000 individuals reached.

Utilizing these materials, CHW/Ps within the community of practice reached over 40,000 individuals between July 2021 and April 2023, via in person outreach door-to-door and in other community settings. Outreach occurred primarily in zip codes with higher concentration of structural inequities (e.g., poverty, uninsurance) in which Latinx, foreign born, and other racial/ethnic minorized groups were overrepresented.

Evaluation surveys for the capacity building workshops revealed high degrees of satisfaction and applicability of the trainings. CHW/Ps rated the content as new, agreed that the workshops helped improve their skills and ability to work with the community, and considered the workshop content to be linguistically and culturally appropriate (Table 1). One promtora shared *“I would have liked to have had all this information from the beginning”* and another shared (regarding motivational interviewing): *“We [CHW/Ps] should never judge them [community members] for their opinions. Just inform and create connections with them, making them feel safe.”*

## 4. Discussion

CHW/Ps have long been a critical workforce in the US for supporting communities disproportionately impacted by a wide range of health issues (6, 25). CHW/Ps were especially critical during the COVID-19 pandemic. Their existing strong and trusting relationships with the communities they serve were crucial for delivering information and resources quickly (14, 26). There was an increase in funding to support CHW/Ps through programs such as CEAL and RAD-x-UP through the National Institutes of Health, as well as local Public Health Departments (17, 27). The increased recognition of their importance and the additional funding provided opportunities to demonstrate CHW/Ps’ effectiveness in promoting health equity in their communities. To effectively realize this opportunity, our research underscored the importance of equipping the CHW/P workforce with resources, support, and capacity building. This was particularly important during the COVID-19 pandemic because information on prevention, diagnosis, and treatment developed rapidly and frequently changed.

### 4.1. Public health implications

The community of practice model was effective for supporting CHW/Ps during the pandemic. In alignment with a CBPR orientation, the community of practice model allowed for shared leadership with CHW/Ps in determining and addressing the needs of the communities they served. CHW/Ps also took a leadership role in designing and implementing evaluation methods. This model provided benefits for partnerships between the academic institution and CBOs, the CHW/Ps, and the local communities. The community of practice model fostered new partnerships and strengthened existing partnerships with the CBOs in which the CHW/Ps were embedded. Partner CBOs received an influx of funds to deploy CHW/Ps to address COVID-19 inequities, however, often did not receive enough resources to sufficiently support the CHW/Ps. The community of practice model enabled the CHW/Ps to come together, prioritize their needs, and academic colleagues could respond directly to those needs. CHW/Ps also benefitted from the community of practice in several ways. They learned from each other’s experiences, skills, and strengths. They were also able to access resources and capacity building opportunities that were not available at their individual organizations. There was high retention of CHW/Ps (93%) in the community of practice, which demonstrated the value that it added to their work. The communities they served also benefitted by gaining access to trusted information and resources related to COVID-19. Given these benefits, the community of practice model could be used for other health issues (e.g., cancer, nutrition, chronic conditions) in the future including addressing housing, food insecurity, and climate inequities. This model also has key implications for investing in the CHW/Ps as a promising public health workforce.

### 4.2. Ongoing and future efforts

The community of practice is poised to take on new challenges. Designed with the community of practice, we have launched a series of CHW/Ps workshops intended to scale capacity development efforts beyond the community of practice. We hosted two series thus far - of three workshops each - for areas exacerbated during the COVID-19 pandemic. One focused on basic needs resources (e.g., housing, food security) and how to link community members and one around mental health resources for children, youth, and adults, as well as coping strategies to prevent burnout among the CHW/Ps. These workshop series were attended by 160 CHW/Ps across three local counties. Moreover, we launched, in partnership with Santa Clara Family Health Plan, a survey (taken by 113 CHW/Ps thus far) and a series of interview with CBO leaders (*n* = 12) with the goal of further assessing existing workforce capacity development programs, challenges, and areas for potential collaboration to promote health equity in marginalized communities. We are also collaborating with a local college offering a CHW certificate to collectively strengthen our local CHW workforce capacity. We plan additional evaluation metrics for these new efforts including: (1) CBOs’ increased capacity to engage in research and academic-community partnerships around health equity issues; and (2) measures of health outcome improvements for community members, as a result of their work with CHW/Ps, by linking to health records of clients at partner CBOs. This includes health records, as well as changes in social determinants of health (e.g., accessing new social services) as a result of their work with CHW/Ps.

Sustainability of this work must also be considered. Transitioning the community of practice to outcomes beyond COVID-19 (e.g., cancer, chronic conditions, dementia) has been a way our team has been able to secure continuous funding. These health outcomes were prioritized by the community of practice due to existing inequities for the Latinx community and are also outcomes for which many groups are looking for partnerships, including local Public Health Departments and Health Plans. Additionally, partnering with groups interested in continuing education for CHW/Ps, especially around certification needs now being considered by many states, can offer additional support for capacity building workshops and similar events our group was already hosting. In terms of group engagement, ensuring any new topics or directions are community driven is crucial to maintain interest and support long-term.

## 5. Conceptual or methodological constraints

Despite the substantial benefits, the community of practice faced numerous challenges. First, the community of practice was primarily focused on providing educational materials, social and peer support, and capacity building. However, there was also a great need for administrative support (e.g., to support subcontracting, infrastructure, and technology). The influx of funding for CHW/Ps during the pandemic was often short on resources for administrative support. Second, information related to prevention, diagnosis, and COVID-19 treatment was constantly changing, requiring ongoing adaptations to educational materials and updated trainings, which was resource-intensive and time consuming. For example, basic epidemiology of COVID-19 evolved into training needs around clinical trial basics, intricacies of testing, addressing vaccine hesitancy and Long-COVID. Third, CBO partners often had limited capacity for tracking metrics, especially given the urgency of the pandemic. Finally, paying CHWs in a timely manner for their work through an academic institution was a challenge. The payment systems in the academic institution are not ideally set up for Spanish-speaking CHW/Ps, for those with diverse documentation status, or for contracting with smaller organizations. There was also a related challenge of securing ongoing and sustainable funding for the community of practice so that it can adapt to meet additional and emerging health equity needs. In systematic reviews of similar programs such as patient navigators for cancer, this has been highlighted as a significant ongoing challenge (28).

Limitation of this work must also be considered. The community of practice model may have been uniquely suited to the pandemic when CBOs were pivoting to address the crisis. With the pandemic subsiding, CBOs may return to prioritizing the health issue most central to their mission. This may make it difficult to bring diverse CHWs together in the future. Additionally, the community of practice is resource-intensive to implement and may be difficult to sustain outside of a global public health crisis.

## 6. Conclusion

The community of practice model proved to be an effective way of supporting CHW/Ps during the pandemic to address the disproportionate burden of the COVID-19 pandemic in our local Latinx community. Our process and impact evaluation demonstrated benefits for community-academic partnerships, for CHW/Ps, and for the communities they serve. This community of practice model can be utilized to address other public health challenges.

## Data availability statement

The raw data supporting the conclusions of this article will be made available by the authors, without undue reservation.

## Ethics statement

The studies involving human participants were reviewed and approved by the Stanford University School of Medicine Institutional Review Board. The patients/participants provided their written informed consent to participate in this study.

## Author contributions

PRE: Conceptualization, Funding acquisition, Investigation, Methodology, Project administration, Supervision, Writing – original draft. YMM: Data curation, Project administration, Visualization, Writing – review & editing. W-tC: Funding acquisition, Project administration, Writing – review & editing. CK: Funding acquisition, Methodology, Resources, Supervision, Writing – review & editing. CT: Methodology, Resources, Writing – review & editing, Data curation, Project administration. MG: Conceptualization, Methodology, Resources, Writing – review & editing. LGR: Conceptualization, Funding acquisition, Methodology, Project administration, Writing – original draft. Promotoras Con Stanford En Acción: Data curation, Resources, Writing – review & editing.
