# Supplementary material for: Community of Practice of Promotoras de Salud to address health inequities during and beyond the COVID-19 pandemic
Source: Front Public Health. 2023 Nov 13;11:1260369. doi: 10.3389/fpubh.2023.1260369 (PMC10679433; doi:10.3389/fpubh.2023.1260369)
Supplement: Supplementary file 1 [file Table_1.docx]

Supplementary Material

Community of Practice of *Promotoras de Salud* to address health inequities during and beyond the COVID-19 pandemic

**Patricia Rodriguez Espinosa, PhD, MPH,*** **Yessica Martinez Mulet, MS, Wei-ting Chen, PhD, Cary Kirk, Mike Gonzalez, MPA, Lisa G. Rosas, PhD, MPH and *Promotoras con Stanford en Acción***

*** Correspondence:** [prespinosa@stanford.edu](mailto:prespinosa@stanford.edu)

Community Health Workers and promotoras’ author group (Promotoras con Stanford en Acción) names and organizations

| **Name** | **Organization** |
| --- | --- |
| Brenda Arenas | Gardner Health Services |
| Nancy Villarreal | Latinas Contra Cancer |
| Isela Luna | Latinas Contra Cancer |
| Macaria Avila | Veggielution |
| Solandyi Aguilar | Veggielution |
| Maria Corrales | Amigos de Guadalupe |
| Martha Gonzalez | Amigos de Guadalupe |
| Imelda Bautista | META, LLC |
| Luz Maria Mendoza | META, LLC |
| Celia Alvarez | De Colores Consulting |
| Angelica Flores | De Colores Consulting |
| Guadalupe Perez | De Colores Consulting |
| Esperanza Garcia | Independent, formerly with Veggielution |
| Teresa Garcia | Independent, formerly with Sí Se Puede Collective |
| Cynthia Colmenares | Independent, formerly with Sí Se Puede Collective |
